# Role of mitogen-activated protein kinase phosphatase-1 in corticosteroid insensitivity of chronic oxidant lung injury

**DOI:** 10.1016/j.ejphar.2014.10.003

**Published:** 2014-12-05

**Authors:** Mariona Pinart, Farhana Hussain, Sima Shirali, Feng Li, Jie Zhu, Andrew R. Clark, Alaina J. Ammit, Kian Fan Chung

**Affiliations:** a Experimental Studies Unit, Airway Disease Section, National Heart and Lung Institute, Imperial College London, London, UK; b Kennedy Institute of Rheumatology Division, Imperial College London, London, UK; c Respiratory Research Group, Faculty of Pharmacy, University of Sydney, NSW, Australia

**Keywords:** Ozone exposure, Emphysema, Lung inflammation, Bronchial hyperresponsiveness, Mitogen-activated protein kinase phosphatase 1 (MKP-1)

## Abstract

Oxidative stress plays an important role in the pathogenesis of chronic obstructive pulmonary disease (COPD) and in the induction of corticosteroid (CS) insensitivity. Chronic ozone exposure leads to a model of COPD with lung inflammation and emphysema. Mitogen-activated protein kinase phosphatase-1 (MKP-1) may underlie CS insensitivity in COPD. We determined the role played by MKP-1 by studying the effect of corticosteroids in wild-type C57/BL6J and MKP-1^−/−^ mice after chronic ozone exposure. Mice were exposed to ozone (3 ppm, 3 h) 12 times over 6 weeks. Dexamethasone (0.1 or 2 mg/kg; intraperitoneally) was administered before each exposure. Mice were studied 24 h after final exposure. In ozone-exposed C57/BL6J mice, bronchial hyperresponsiveness (BHR) was not inhibited by both doses of dexamethasone, but in MKP-1^−/−^ mice, there was a small inhibition by high dose dexamethasone (2 mg/kg). There was an increase in mean linear intercept after chronic ozone exposure in both strains which was CS-insensitive. There was lesser inflammation after low dose of dexamethasone in MKP-1^−/−^ mice compared to C57/Bl6J mice. Epithelial and collagen areas were modulated in ozone-exposed MKP-1^−/−^ mice treated with dexamethasone compared to C57/Bl6J mice. MKP-1 regulated the expression of MMP-12, IL-13 and KC induced by ozone but did not alter dexamethasone׳s effects. Bronchial hyperresponsiveness, lung inflammation and emphySEMa after chronic exposure are CS-insensitive, and the contribution of MKP-1 to CS sensitivity in this model was negligible.

## 1 Introduction

Oxidative stress is a feature of the airways and lungs of patients with chronic obstructive pulmonary disease (COPD), resulting from the release of reactive oxygen and nitrogen species from inflammatory and immune cells in the airways and from the direct exposure to oxidants present in cigarette smoke or environmental pollutants (Chung and Marwick, 2010). Oxidative stress plays an important role in the pathogenesis of COPD since cigarette smoke and particulate exposure are potent inducers of oxidative stress (Repine et al., 1997, Risom et al., 2005, Chung and Adcock, 2008). The mechanisms and pathways by which oxidative stress can lead to chronic inflammation and emphysema have been investigated in mouse models of cigarette exposure (Shapiro, 2007, Taraseviciene-Stewart and Voelkel, 2008). Thus, the importance of oxidative stress in inducing emphysema has been demonstrated in nuclear factor-E2-related factor-2 (Nrf2) knockout mice, which, through their diminished capacity to mount antioxidant defences, develop increased susceptibility to emphysema and lung inflammation following cigarette smoke exposure (Rangasamy et al., 2004). Furthermore, direct exposure of mice to an oxidant gas, ozone, results in emphysema and chronic lung inflammation reminiscent of COPD (Triantaphyllopoulos et al., 2011). Oxidant stress also causes bronchial hyperresponsiveness resulting from an increase in contractility of the airways (Li et al., 2011).

Corticosteroids (CSs) are widely used in the treatment of chronic airway inflammatory diseases. Although they are the most potent anti-inflammatory agent used in the treatment of asthma, they are not always effective as in patients with severe asthma and COPD. Several mechanisms may underlie CS insensitivity (Adcock et al., 2008), which includes the role of the mitogen-activated protein kinases (MAPK) (Chung, 2011). MAPK phosphatases terminate MAPK activation by dephosphorylating both threonine and tyrosine residues (Camps et al., 2000). MKP-1 or DUSP-1, the founding member of this group of at least 10 phosphatases, is an effective inhibitor of JNK and p38 MAPK. MKP-1 is up-regulated by oxidative stress and other stimuli such as ultra-violet light, TNFα, IL-1 and several toll-receptor ligands (Keyse, 2000). MKP-1 may be involved in corticosteroid responses since corticosteroids inhibit p38 MAPK in macrophages from MKP-1^+/+^ mice but not in those from MKP-1^−/−^ littermates where there was an inability of corticosteroids to inhibit cytokine release from macrophages of these mice (Abraham et al., 2006). Thus, MKP-1 expression may underlie CS insensitivity found in various respiratory diseases such as severe asthma and COPD (Chung, 2011).

In order to investigate whether MKP-1 can modulate the effects of oxidative stress and their responses to CS, we used a model of chronic exposure to ozone that leads to alveolar space enlargement and destruction together with a chronic inflammatory process (6). Although CS have been shown to inhibit the effects of a single exposure to ozone (Salmon et al., 1998, Toward and Broadley, 2002), it is not known whether they inhibit the effect of multiple exposures to ozone. We therefore examined the effect of CS in a chronic ozone model, and determined the role played by MKP-1 by studying the MKP-1^−/−^ mouse.

## 2 Materials and methods

### 2.1 Mice

Pathogen-free, 10–12 week old male C57/BL6J mice (Harlan, UK) and gender-matched MKP-1^−/−^ mice (Kennedy Institute, Imperial College, UK) were housed within ‘maximiser’ filter-topped cages (Maximiser, Theseus caging system Inc., Hazelton, PA, USA). The original MKP-1 null strain provided by Bristol Myers Squibb (Dorfman et al., 1996) was on a mixed C57/BL6J-129Sv genetic background. This strain was back-crossed against C57/BL6J over nine generations, then intercrossed heterozygotes and MKP-1^+/+^ and MKP-1^−/−^ mice were identified by PCR-based screen of genomic DNA from tail snips. MKP-1^−/−^ colonies are identical in genetic background (almost pure C57/BL6J), but differ only at the MKP-1 locus. We therefore used wild type C57/BL6J as controls. The protocols were approved by the Imperial College Biosciences group and performed under a license from the Home Office UK government.

### 2.2 Study design and methods

The experiments were performed within the legal framework of the United Kingdom under a Project License granted by the Home Office of Her Majesty׳s government. The researchers hold Personal Licenses to perform the experiments described here. MKP-1^−/−^ and C57/BL6J were investigated and received ozone twice a week for a period of 6 weeks (a total of 12 exposures). Ozone was generated from an ozoniser (Model 500 Sander Ozoniser, Germany), mixed with air for 3 h at 2.5 parts per million (ppm) in a sealed Perspex container. Control animals received medical air only over the equivalent period. Ozone concentration was continuously monitored with an ozone probe (ATi Technologies, Ashton-U-Lyne, UK). Ozone exposure was carried out in 3 groups: (i) ozone and vehicle, (ii) ozone and 0.1 mg/kg dexamethasone and (iii) ozone and 2 mg/kg dexamethasone. During the final 4 weeks of ozone exposure, animals received either dexamethasone or vehicle 2 h prior to each exposure to ozone (i.e. 8 injections in all). After 2 weeks of ozone exposures, mice received intraperitoneal (i.p.) injections of either 0.1 mg/kg or 2 mg/kg dexamethasone (D4902-1G Sigma Aldrich, USA) dissolved in 0.1 ml Dulbecco phosphate buffered saline (PBS) (Sigma, Dorset, UK). One group exposed to ozone received the same volume (0.1 ml) of PBS as vehicle. Mice were studied 24 h after the last exposure to ozone.

### 2.3 Measurement of bronchial hyperresponsiveness

24 h following exposure, mice were anesthetized with an intraperitoneal injection of anesthetic solution containing midazolam (Roche Products Ltd., Welwyn Garden City, UK) and Hypnorm (0.315 mg/ml fentanyl citrate and 10 mg/ml fluanisone; Janssen Animal Health, Wantage, UK). Mice were tracheostomized and ventilated (Mini Vent type 845, Hugo Sach Electronic, Germany; rate: 250 breaths/min and tidal volume: 250 μl). Mice were monitored in a whole body plethysmograph with a pneumotachograph connected to a transducer (EMMS, Hants, UK). Transpulmonary pressure was assessed via an esophageal catheter. Instantaneous calculation of pulmonary resistance (*R*_L_) was obtained. Increasing concentrations of acetylcholine chloride (ACh) (Sigma, Dorset, UK) (4–256 mg/ml) were administered with an Aeroneb^®^ Lab Micropump Nebulizer (EMMS, Hants, UK), and *R*_L_ was recorded for a 3-min period following each concentration. *R*_L_ after each concentration was expressed as percentage change from baseline *R*_L_ measured following nebulized PBS (Sigma, Dorset, UK). The concentration of acetylcholine required to increase *R*_L_ by 100% from baseline was calculated (PC_100_).

### 2.4 Measurement of inflammation and mean linear intercept, *L*_m_

Following an overdose of pentobarbitone anesthetic, the lungs were dissected out and were inflated by injecting fresh 4% paraformaldehyde into the lungs to provide 25 cm of water pressure for at least 4 h. Lungs were processed using a histological automatic tissue processor and embedded in paraffin. Paraffin blocks were sectioned to expose the maximum surface area of lung tissue in the plane of the bronchial tree. 5 μm sections cut and stained with haematoxylin and eosin and Masson׳s trichrome stain were point-counted to assess morphological changes of airway epithelium, collagen deposition and airway smooth muscle (ASM) mass.

The mean linear intercept (*L*_m_) is a measure of the surface area-to-volume ratio representing a stereological metric of alveolar size. Using a reticule with a Thurlbeck grid comprising 5 lines (each 550 μM long) and 10 fields per section was assessed at random. Fields with airways or vessels were avoided by moving one field in any one direction. The total score for each section was determined by counting the number of times the alveolar wall tissue intercepted each line. *L*_m_ was calculated by dividing the length of the line by the number of tissue intercepts counted.

The inflammatory response observed in the haematoxylin–eosin-stained lung sections was scored on a 0–3 scale as follows: 0=no inflammatory response, 1=mild inflammation with foci of inflammatory cells in bronchial or vascular wall and in alveolar septa; 2=moderate inflammation with patchy inflammation or localized inflammation in walls of bronchi or blood vessel and alveolar septa and less than 1/3 of lung cross-sectional area is involved; and 3=severe inflammation with diffuse inflammatory cells in walls of bronchi or blood vessels, and alveoli septa; between one third to two thirds of the lung area is involved.

All counts on histology sections were performed by one investigator who was unaware of the treatment protocol.

### 2.5 Reverse transcription, and real-time PCR of mouse lungs

RNA was extracted from frozen stored lung tissue using an RNeasy Mini kit (Qiagen). RNA yield was then amplified via PCR using an Omniscript Reverse Transcriptase kit (Qiagen) and stored at −80 °C until required. 0.5 μg per sample of RNA was used to synthesize single-stranded complementary DNA (cDNA) using random hexamers and an avian myeloblastosis virus reverse transcriptase (Promega). The cDNA generated was used as a template in subsequent real-time PCR analyses to determine transcript levels by using Rotor Gene (Rotor Gene 3000, Corbett Research) and QuantiTech SYBR Green PCR Master Mix Reagent (Qiagen). Sequences of forward and reverse primers for MMP-12, IFNγ, IL-13 and KC were designed using Primer Express software (version 2; Applied Biosystems, Foster City, CA). Cycling conditions were as follows: step 1, 15 min at 95 °C; step 2, 20 s at 94 °C; step 3, 20 s at 55 °C; step 4, 20 s at 72 °C, repeating step 2 to step 4, 55 times. Gene expression was expressed as a ratio of the gene of interest mRNA to GAPDH mRNA.

### 2.6 Data analysis

Data are presented as mean±S.E.M. Those variables following a normal distribution were submitted to one-way ANOVA analysis of variance followed by Bonferroni post-hoc testing for comparisons between two individual groups. Otherwise, Kruskal–Wallis test was used followed by Mann–Whitney–Wilcoxon test for comparisons between two individual groups. Statistical significance was accepted at *P*-value less than 0.05.

## 3 Results

### 3.1 Effect of dexamethasone on chronic-exposed ozone-induced BHR

**Fig. 1:**
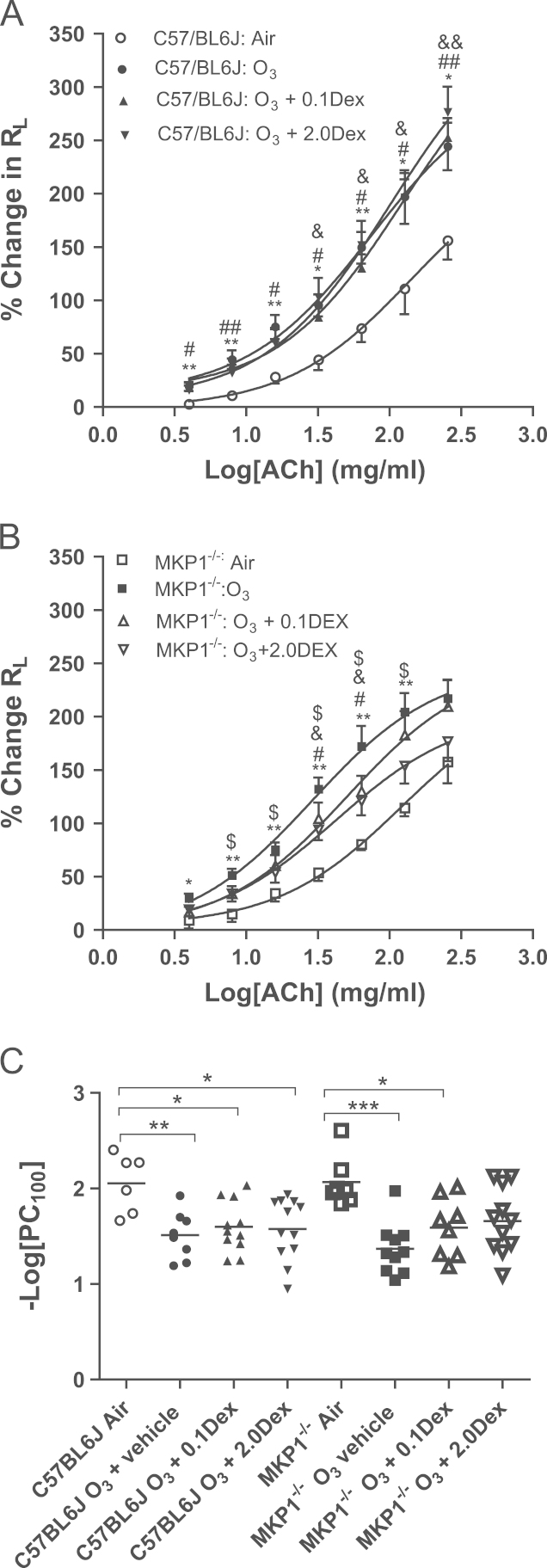
Airway hyperresponsiveness. Concentration–response curves to acetylcholine (ACh) (Panels A and B) and log provocative concentration of ACh required to increase lung resistance (*R*_L_) by 100% from baseline (log PC_100_) (Panels C) of C57/BL6J and MKP-1^−/−^ mice exposed to air or to multiple ozone exposures over 6 weeks. Data expressed as mean±S.E.M. Horizontal bars indicate mean. For Panels A and B, ^*^P<0.05; ^**^P<0.01: ozone compared to air-exposed mice; ^≠^*P*<0.05; ^≠≠^*P*<0.01, ozone-exposed and treated with 0.1 mg/kg dexamethasone compared to air-exposed mice; *P*<0.05; *P*<0.01: ozone-exposed and treated with 2 mg/kg dexamethasone compared to air-exposed mice; ^$^*P*<0.05: ozone-exposed mice treated with saline (vehicle) compared to ozone-exposed and treated with 2 mg/kg dexamethasone. For Panel C, ^*^P<0.05; ^**^P<0.01; ^***^P<0.001.

Following ozone exposure, there was an increase in BHR in all groups of mice compared to control in both strains (Fig. 1A and B). In MKP-1^−/−^ mice, ozone caused an increase in *R*_L_ compared to air-exposed mice, at all doses except for the highest concentration (256 mg/ml) of ACh, and at the intermediate concentrations of 32 and 64 mg/ml for the groups treated with either 0.1 mg/kg dexamethasone or 2 mg/kg dexamethasone (Fig. 1B). Furthermore, ozone-exposed MKP-1^−/−^ mice treated with vehicle showed a significant increase in *R*_L_ compared to 2 mg/kg dexamethasone-treated mice, at concentrations between 8 and 128 mg/ml ACh. When comparing between strains, significant differences were seen at the highest dose of ACh in mice administered the highest dose of dexamethasone (*P*<0.01).

When bronchial responsiveness was assessed as PC_100_, the decrease in PC_100_ was not inhibited by the two doses of dexamethasone in C57/Bl6J mice (Fig. 1C). In MKP-1^−/−^ mice, there was still a significant reduction in PC_100_ after ozone exposure after 0.1 mg/kg dexamethasone but not after the 2 mg/kg dose indicating a potential reversal of ozone induced BHR. Thus, in both strains, there was induction of corticosteroid insensitivity, but MKP-1^−/−^ mice compared to C57/BL6J mice responded partially to high-dose dexamethasone.

### 3.2 Lung inflammation scores

**Fig. 2:**
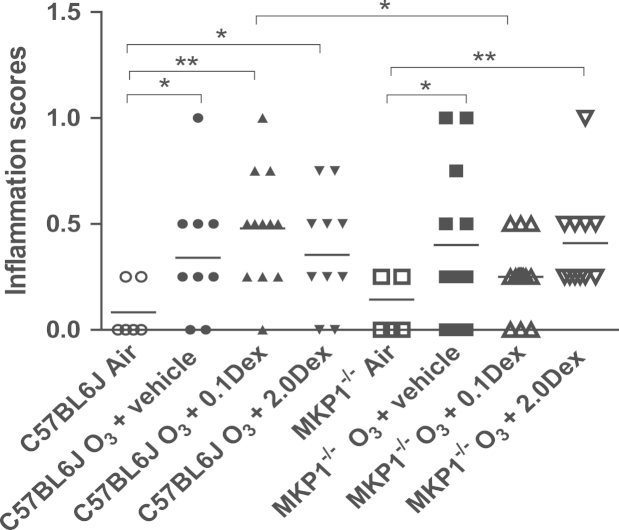
Lung inflammation. Representative light microscopic lung sections from C57/Bl6 and MKP-1^−/−^ mice showing the inflammatory response in the lungs following exposure to ozone alone (Panels B and E) and the effect of high dose dexamethasone (Panels C and F), compared to air-exposed mice (panels A and D). Bar in Panel F is 10 μM. Panel G shows the individual inflammation scores in the lungs of C57/BL6J and MKP-1^−/−^ mice exposed to ozone over 6 weeks. ^*^P< 0.05; ^**^P<0.01.

Ozone-exposed groups of C57/BL6J mice showed an increase in inflammation scores with peribronchial and perivascular inflammatory cell infiltrates in lung sections compared to air-exposed mice, particularly in the group treated with 0.1 mg/kg dexamethasone (*P*<0.01, Fig. 2). Similar findings were observed in the MKP1^−/−^ mice, expect for the group treated with low dose dexamethasone that led to lower lung inflammation scores. Lower inflammatory scores were observed in the ozone-exposed MKP-1^−/−^ mice treated with 0.1 mg/kg dexamethasone compared to the C57/BL6J mice (*P*<0.05).

### 3.3 Mean linear intercept, *L*_m_

**Fig. 3:**
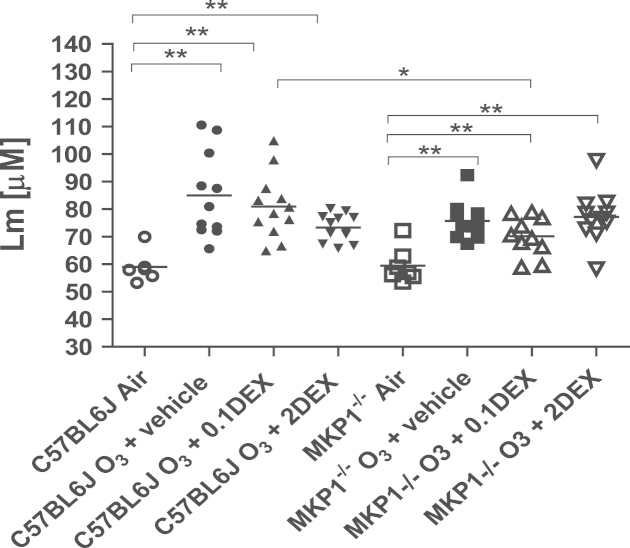
Mean linear intercept (*L*_m_). Representative light microscopic lung sections from C57/Bl6 and MKP-1^−/−^ mice showing alveolar damage and enlargement in the lungs following exposure to ozone alone (Panels B and E) and the effect of high dose dexamethasone (Panels C and F), compared to air-exposed mice (Panels A and D). Bar in Panel F is 20 μM. Panel G shows the individual *L*_m_ values measured in lung sections of C57/BL6J and MKP-1^−/−^ mice exposed to ozone over 6 weeks. Horizontal bar indicates mean values. ^*^P< 0.05; ^**^P<0.01.

*L*_m_ increased in C57/BL6J and MKP-1^−/−^ ozone-exposed mice compared to air-exposed mice (both *P*<0.01, compared to unexposed mice) (Fig. 3). *L*_m_ was still increased in both strains after ozone exposure following dexamethasone pretreatment at both doses. However, MKP-1^−/−^ mice exposed to ozone and receiving the low dose dexamethasone had a lower *L*_m_ when compared to ozone-exposed C57/Bl6J mice (*P*<0.05).

### 3.4 Effect on epithelial, airway smooth muscle and collagen areas

**Fig. 4:**
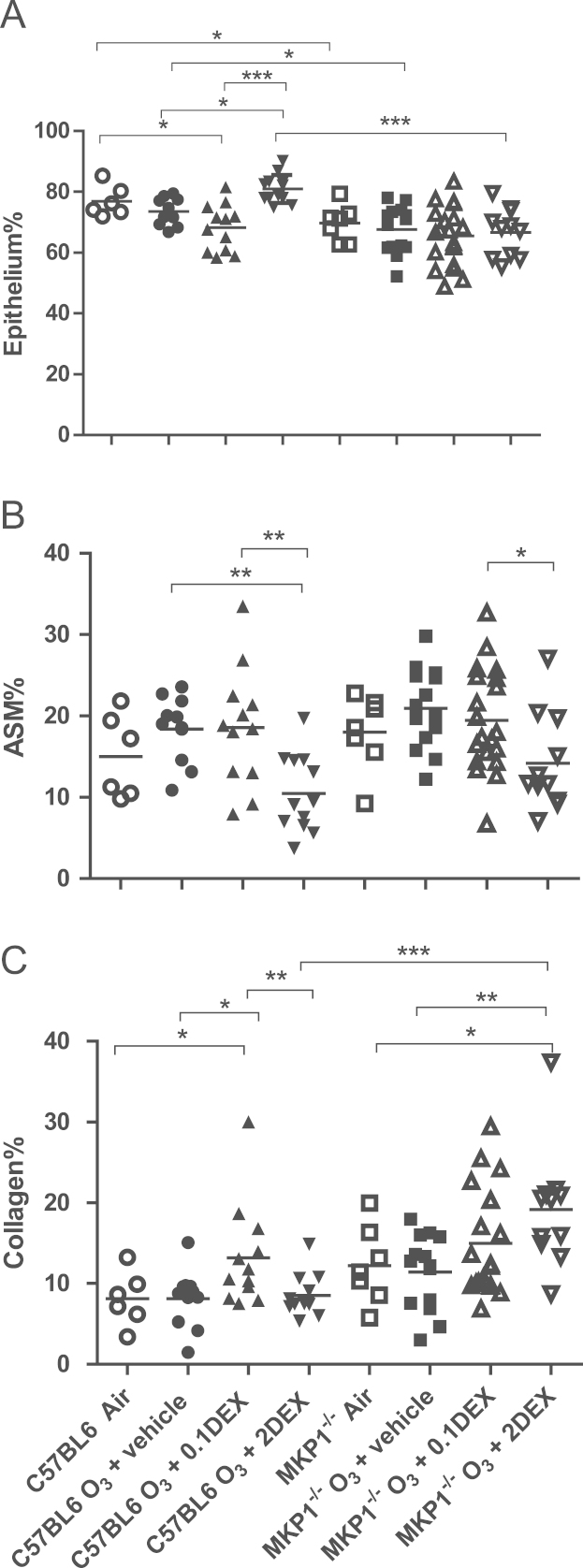
Changes in airway wall. Individual and mean% of area of bronchial wall occupied by epithelium (Panel A), airway smooth muscle (ASM) (Panel B) and collagen (Panel C) in C57/BL6J MKP-1^−/−^ mice after chronic exposure to ozone or air, as determined by point-counting. The ozone-exposed mice were treated with saline (vehicle) or with 0.1 mg/kg or 2.0 mg/kg dexamethasone (Dex) prior to each exposure. ^*^P< 0.05; ^**^P<0.01; ^***^P<0.001.

At baseline, MKP-1^−/−^ mice showed a significantly lower epithelial area compared to C57/Bl6J mice (Fig. 4A). High dose dexamethasone increased epithelial area in C57/BL6J ozone-exposed mice, an effect not seen in MKP-1^−/−^ mice, indicating control of epithelial cell area by dexamethasone dependent on MKP-1. In C57/BL6J mice, the high dose of dexamethasone decreased airway smooth muscle area, an effect that persisted in MKP-1^−/−^ mice (Fig. 4B). High dose dexamethasone while having no effect on collagen area in C57/BL6J ozone-exposed mice increased it in MKP-1^−/−^ ozone-exposed mice (Fig. 4C). Thus, there was a role for MKP-1 in modulating the effect of dexamethasone on the epithelial and collagen surface area following ozone exposure.

### 3.5 Effect on gene expression of MMP-12, IFNγ and IL-13

Baseline mRNA expression of MMP-12, KC and IL-13, but not of IFNγ, was increased in MKP-1^−/−^ mice (Table 1).

**Table 1:** Inflammatory gene expression in the lungs of C57/Bl6J and MKP-1^−/−^ mice.

**Inflammatory gene**	**C57/BL6J**	**MKP-1**^−/−^	***P*****value**
MMP-12	1.783±0.556	10.012±1.566	<0.001
IL-13	3.518±1.832	85.142±26.492	0.010
IFN-γ	1.770±0.996	18.776±10.094	NS
KC	1.345±0.356	5.397±1.692	0.021

*n*=6 in each group. Results shown as mean ±S.E.M.

#### 3.5.1 MMP-12

**Fig. 5:**
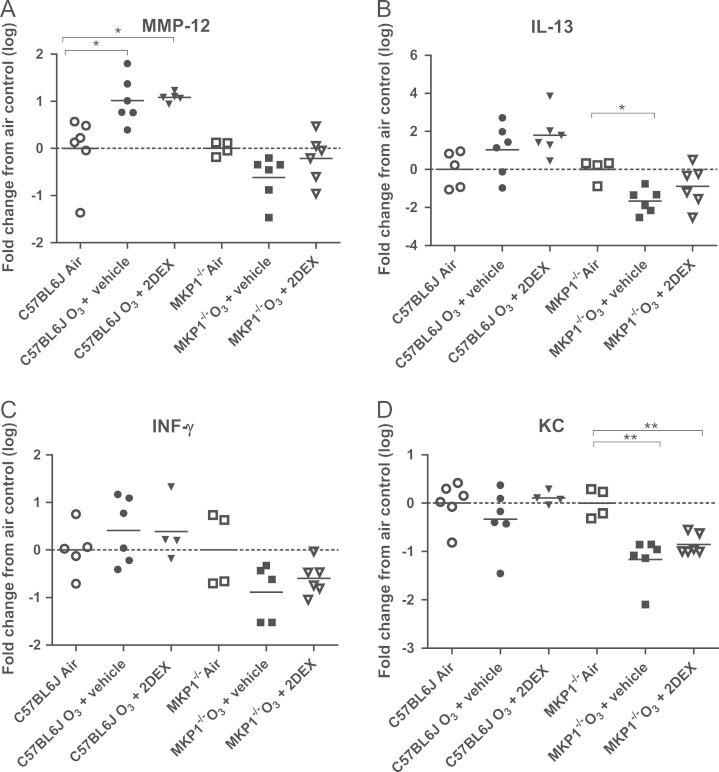
Lung expression of inflammatory genes. Individual and mean fold-change from air control for gene expression of MMP-12 (Panel A), IL-13 (Panel B), IFNγ (Panel C), and KC (Panel D) in lungs of mice exposed to either air or ozone (O_3_) or to ozone but pre-treated with 2.0 mg/kg dexamethasone (DEX) in C57/BL6J and in MKP-1^−/−^ mice following chronic exposure to ozone. For each strain, the data has been normalized to expression in air-exposed mice alone. Bars show mean. ^*^P< 0.05; ^**^P<0.01.

In ozone-exposed C57/BL6J mice, there was an enhanced expression of MMP-12 after ozone exposure (*P*<0.05, Fig. 5A) and dexamethasone (2.0 mg/kg) had no effect on this increased MMP-12 expression. By contrast, in MKP-1^−/−^ mice, there was no effect of ozone exposure and neither did dexamethasone have an effect.

#### 3.5.2 IL-13

There was no effect of dexamethasone on the expression of IL-13 in ozone-exposed C57/BL6J but there was a reduction in the expression of IL-13 in MKP-1^−/−^ mice (*P*<0.05, Fig. 5B).

#### 3.5.3 IFNγ

There was no effect of ozone or of dexamethasone on IFNγ expression in both strains (Fig. 5C).

#### 3.5.4 KC

In C57/BL6J mice, there was no effect of ozone exposure or of ozone and dexamethasone treatment on the expression of KC. However, in MKP1^−/−^ mice, KC gene expression was reduced after ozone exposure, but this was not affected by dexamethasone (Fig. 5D).

## 4 Discussion

We determined the role of MKP-1 on the effects of corticosteroids in a chronic model of ozone exposure of chronic lung inflammation and emphysema (Triantaphyllopoulos et al., 2011). We used a well-documented MKP-1 knock-out mouse (Dorfman et al., 1996) whose macrophages demonstrate CS insensitivity (Abraham et al., 2006) to determine whether the knock-down of MKP-1 would affect, first, the response of the lungs to chronic ozone exposure and secondly, the effect of dexamethasone on the response of the lungs to ozone. The overall findings of the study are now summarized in Table 2. Overall, dexamethasone had no effect in reversing the bronchial hyperresponsiveness, mean linear intercept and inflammation, and the expression of the emphysema-related genes, MMP-12, IL-13 and IFNγ, and the neutrophil-associated gene, KC induced by ozone. Some effects of dexamethasone were observed on epithelial and airway smooth muscle areas but these were not affected by ozone exposure. In the light of previous studies where CS suppressed BHR and lung inflammation caused by single exposure to ozone (Salmon et al., 1998, Toward and Broadley, 2002), we conclude that the chronic ozone exposure led to a state of CS insensitivity. In addition, we have also shown that in C65/BL6 mice the effect of a single exposure to ozone is blocked by dexamethasone, in contrast to the multiple exposure model (unpublished data). In addition to BHR and lung inflammation, the emphysematous process and the expression of emphysema-related genes are also insensitive to CS.

**Table 2:** Summary of involvement of MKP-1 on the effects of ozone and on the effects of dexamethasone on ozone׳s effect.

**Parameter**	**Effect of ozone exposure**	**Effect MKP-1 on ozone**	**Effect of dexamethasone on ozone**׳**s effects**	**Effect of MKP-1 on dexamethasone**׳**s effect on ozone**
**Bronchial hyperrresponsiveness**	↑	0	0	↓ (hd)^a^
**Mean linear intercept,*****L***_**m**_	↑	0	0	0
**Lung inflammatory score**	↑	0	0	↓ (ld)^b^
**Epithelial area (%)**	0	0	↑ (hd)^a^	↓ (ld)^b^
**Airway smooth muscle area (%)**	0	0	↓ (hd)^a^	0
**Collagen area (%)**	0	0	↑ (ld)^b^	↓ (hd)^a^
**MMP-12 gene expression**	↑	↑	0	0
**IL-13 gene expression**	0	↑	0	0
**IFN γ gene expression**	0	0	0	0
**KC gene expression**	0	↑	0	0

a hd: high dose dexamethasone (2 mg/kg).

b ld: low dose dexamethasone (0.1 mg/kg).

We found that MKP-1 had a little effect on BHR, inflammation and emphysema induced by chronic ozone exposure (Table 2). High dose dexamethasone caused some reversal of BHR induced by chronic exposure to ozone in MKP-1^−/−^ mice indicating that MKP-1 may partly contribute to the CS insensitivity. There was also some reversal of the lung inflammatory scores observed with low dose but not high dose dexamethasone in MKP-1^−/−^ mice. The lack of effect of dexamethasone on the emphysema as measured by the increase in mean linear intercept, *L*_m_, induced by chronic ozone exposure was also observed in MKP-1^−/−^ mice, indicating that MKP-1 is neither involved in the induction of emphysema nor in the effect of corticosteroids on emphysema. While ozone exposure did not change epithelial, airway smooth muscle or collagen areas, dexamethasone while decreasing ASM area, increased epithelial and collagen areas. MKP-1 modulated these changes in epithelial and collagen areas induced by corticosteroids.

There are various indications that MKP-1 could be involved in controlling the inflammatory response to oxidants. MKP-1 deficiency is associated with an increase in the production of many cytokines including TNF-α, IL-6, IL-1β (Chen et al., 2002, Shepherd et al., 2004, Zhao et al., 2005), which is in agreement with the large increase in gene expression of KC, IL-13 and MMP-12 in the lungs of unexposed MKP-1^−/−^ mice compared to wild-type mice. Under baseline conditions, MKP-1 may act as a brake on the expression of various inflammatory cytokines possibly through down-regulating the activation of MAPKs such as p38 and JNK in the lungs. However, the regulation of various genes under conditions of oxidative stress is more complex. On chronic exposure to ozone, the expression of MMP-12, IL-13 and KC was down-regulated in MKP-1^−/−^ mice, indicating that MKP-1 may prevent the inhibition of oxidative stress-induced genes in the lung. MKP-1 did not contribute to the modulation of dexamethasone on the expression of MMP-12, IL-13 and KC under conditions of oxidative stress.

A lack of MKP-1 has been proposed in the genesis of corticosteroid insensitivity. Corticosteroids are known to induce MKP-1 in pulmonary tissues (Kelly et al., 2012), in bronchoalveolar macrophages (Bhavsar et al., 2008) and in human airway epithelial cells (Kaur et al., 2008). The muted induction of MKP-1 expression in alveolar macrophages of severe asthmatics is correlated with corticosteroid insensitivity of these cells (Bhavsar et al., 2008). There is some evidence that MKP-1 may be essential for the effect of CS, and a deficiency of MKP-1 may cause corticosteroid insensitivity. Thus, glucocorticoid receptor activation appears to be essential for corticosteroid-mediated MKP-1 induction in macrophages (Bhattacharyya et al., 2007), since there are putative glucocorticoid response elements (GRE) binding sites on their promoter regions (Tchen et al., 2010). MKP-1 may be involved in corticosteroid responses since corticosteroids inhibit p38 MAPK in macrophages from MKP-1^+/+^ mice but not in those from MKP-1^−/−^ littermates; this was accompanied by an inability of corticosteroids to inhibit cytokine release from macrophages of MKP-1^−/−^ mice (Abraham et al., 2006). Previous studies performed in bone-marrow derived macrophages from MKP-1^−/−^ mice have reported glucocorticoid-mediated inhibition of phosphorylation of p38 MAPK but not of ERK-1 and ERK-2 (Maier et al., 2007). Studies in our MKP-1 model indicate that MKP-1 would prevent the expression of ozone-induced cytokines and partly improve the response of BHR and lung inflammation to CS, contrary to our expectations.

One potential explanation for these findings relate to the possibility that MKP-1 is inactivated by oxidation leading to greater susceptibility to proteasome degradation after chronic exposure (Kamata et al., 2005). The redox state of MKP-1 may determine the response of the lungs to ozone, particularly after chronic oxidative stress of ozone exposure, such that the chronic-exposed mice would behave like a partially-depleted MKP-1 mouse. On the other hand, MKP-1 can be phosphorylated by MAPKs such as ERK, which protects MKP-1 from proteasome-mediated degradation (Sohaskey and Ferrell, 2002). In addition, ERK can mediate the induction of MKP-1 in macrophages. Thus, the level of activity of ERK and possibly of other kinases may determine in turn the activity of MKP-1.

### 4.1 Conclusion

We have shown that chronic exposure to ozone leads to a corticosteroid-insensitive model of BHR, emphysema and lung inflammation. MKP-1 may increase the expression of MMP-12, IL-13 and KC in the presence of ozone exposure, but is not involved in the effect of corticosteroids. However, MKP-1 may modulate to a small extent the effects of corticosteroids on ozone induction of BHR, and inflammation, with a greater contribution to the epithelial and collagen changes in the airways, representative of the remodelling process.
